# The founding charter of the Omic Biodiversity Observation Network (Omic BON)

**DOI:** 10.1093/gigascience/giad068

**Published:** 2023-08-26

**Authors:** Raïssa Meyer, Neil Davies, Kathleen J Pitz, Chris Meyer, Robyn Samuel, Jane Anderson, Ward Appeltans, Katharine Barker, Francisco P Chavez, J Emmett Duffy, Kelly D Goodwin, Maui Hudson, Margaret E Hunter, Johannes Karstensen, Christine M Laney, Margaret Leinen, Paula Mabee, James A Macklin, Frank Muller-Karger, Nicolas Pade, Jay Pearlman, Lori Phillips, Pieter Provoost, Ioulia Santi, Dmitry Schigel, Lynn M Schriml, Alice Soccodato, Saara Suominen, Katherine M Thibault, Visotheary Ung, Jodie van de Kamp, Elycia Wallis, Ramona Walls, Pier Luigi Buttigieg

**Affiliations:** HGF MPG Joint Re­search Group for Deep-Sea Eco­logy and Tech­no­logy, Alfred Wegener Institute, Helmholtz Centre for Polar and Marine Research, Bremerhaven 27570, Germany; Faculty of Geosciences, University of Bremen, Bremen 28359, Germany; HGF MPG Joint Re­search Group for Deep-Sea Eco­logy and Tech­no­logy, Max Planck Institute for Marine Microbiology, Bremen 28359, Germany; Gump South Pacific Research Station, University of California Berkeley, Moorea 98728, French Polynesia; Berkeley Institute for Data Science, University of California, Berkeley, CA 94720, USA; Science Department, Monterey Bay Aquarium Research Institute, Moss Landing, CA 95039, USA; Department of Invertebrate Zoology, National Museum of Natural History, Smithsonian Institution, Washington, DC 20560, USA; School of Ocean and Earth Science, University of Southampton, Southampton SO17 1BJ, UK; Ocean Technology and Engineering Group, National Oceanography Center, Southampton SO14 3ZH, UK; Department of Anthropology, New York University, New York City, NY 10012, USA; Intergovernmental Oceanographic Commission of UNESCO, Ocean Biodiversity Information System, Oostende 8400, Begium; Global Genome Biodiversity Network Secretariat Office, National Museum of Natural History, Smithsonian Institution, Washington, DC 20560, USA; Science Department, Monterey Bay Aquarium Research Institute, Moss Landing, CA 95039, USA; Tennenbaum Marine Observatories Network and MarineGEO Program, Smithsonian Environmental Research Center, Edgewater, MD 21037, USA; National Oceanic & Atmospheric Administration, NOAA Ocean Exploration, La Jolla, CA 92037, USA; Te Kotahi Research Institute, University of Waikato, Hamilton 3240, New Zealand; Wetland and Aquatic Research Center, U.S. Geological Survey, Gainesville, FL 32653, USA; Department of Physical Oceanography, GEOMAR Helmholtz Centre for Ocean Research Kiel, Kiel 24105, Germany; Science department, National Ecological Observatory Network, Boulder, CO 80301, USA; Geosciences Research Division, Scripps Institution of Oceanography, La Jolla, CA 92093, USA; Observatory Leadership department, National Ecological Observatory Network, Boulder, CO 80301, USA; Botany and Biodiversity Informatics, Agriculture and Agri-Food Canada (AAFC), Ottawa, Ontario K1A 0C6, Canada; College of Marine Science, University of South Florida, St. Petersburg, FL 33701, USA; European Marine Biological Resource Centre (EMBRC-ERIC), Paris 75252, France; IEEE, Paris 75116, France; Agriculture and Agri-Food Canada (AAFC), Harrow N0R 1G0, Ontario, Canada; Intergovernmental Oceanographic Commission of UNESCO, Ocean Biodiversity Information System, Oostende 8400, Begium; European Marine Biological Resource Centre (EMBRC-ERIC), Paris 75252, France; Hellenic Centre for Marine Research (HCMR), Institute of Marine Biology, Biotechnology and Aquaculture (IMBBC), Heraklion GR71003, Greece; GBIF | Global Biodiversity Information Facility, Copenhagen DK-2100, Denmark; Epidemiology & Public Health, University of Maryland School of Medicine, Baltimore, MD 21201, USA; European Marine Biological Resource Centre (EMBRC-ERIC), Paris 75252, France; Intergovernmental Oceanographic Commission of UNESCO, Ocean Biodiversity Information System, Oostende 8400, Begium; Science department, National Ecological Observatory Network, Boulder, CO 80301, USA; CNRS-MNHN-SU-EPHE-UA, 75005 Paris, France; CSIRO Environment, Hobart 7004 Tasmania, Australia; CSIRO, Melbourne 3071, Australia; Data Science department, Critical Path Institute, Tucson, AZ 85718, USA; HGF MPG Joint Re­search Group for Deep-Sea Eco­logy and Tech­no­logy, Alfred Wegener Institute, Helmholtz Centre for Polar and Marine Research, Bremerhaven 27570, Germany; Information, Data and Computer Center, Helmholtz Metadata Collaboration/GEOMAR Helmholtz Centre for Ocean Research Kiel, Kiel 24105, Germany

**Keywords:** biodiversity, omics, eDNA, earth observation, GEO BON, essential variables, biomonitoring

## Abstract

Omic BON is a thematic Biodiversity Observation Network under the Group on Earth Observations Biodiversity Observation Network (GEO BON), focused on coordinating the observation of biomolecules in organisms and the environment. Our founding partners include representatives from national, regional, and global observing systems; standards organizations; and data and sample management infrastructures. By coordinating observing strategies, methods, and data flows, Omic BON will facilitate the co-creation of a global omics meta-observatory to generate actionable knowledge. Here, we present key elements of Omic BON's founding charter and first activities.

## Background

### Omics biodiversity observation - challenge and opportunity

All life on earth - from microbes to giant sequoias - depends on biomolecules involved in the transfer of information, such as genes, transcripts, and proteins. Analysis of these biomolecules can help us monitor biodiversity and understand how it is changing in response to human activities, environmental or biological pressures, or due to genetic drift or mutations. Molecular techniques, collectively known as *omics*, offer a powerful toolset for such investigations.

Referring to the holistic study of elements that compose a greater whole, *omics* is used by the biomolecular community to describe the study of DNA and RNA sequences, proteins, metabolites, and other biomolecules in (i) organisms (genomics, transcriptomics, proteomics, or metabolomics) or (ii) environmental material samples (environmental DNA/RNA, metagenomics, metatranscriptomics, metaproteomics, and metabolomics analyses).

Widely applied in biomedical research, omics has great potential in environmental and biodiversity studies [1, 2]. Various sociotechnical challenges, however, continue to impede the use of biomolecular evidence in public policy and resource management options [3, 4]. The challenges to be surmounted include (i) insufficient coordination among the various actors involved in biodiversity omics; (ii) sparse and infrequent omic observations across many regions, environments, or ecosystems, especially with respect to baseline and time-series data; (iii) insufficient convention and agreement on common practices for accessing, tracking, and storing the biosamples that underpin omic analyses; and (iv) a lack of standardized practices and operationalization of FAIR (Findable, Accessible, Interoperable, Reusable) [5] and CARE (Collective Benefit, Authority to Control, Responsibility, and Ethics) [6] data principles. Taken together, these obstacles challenge the seamless integration of omic observations in scientific syntheses and can have significant ethical, legal, and social implications (e.g., contributing to the limited success of Convention on Biological Diversity (CBD) Access & Benefit Sharing (ABS) provisions [7]).

Addressing these challenges will require global coordination of key actors; overcoming regional silos by ensuring compatibility of baselines, time-series, and reference libraries; developing concerted strategies and agreed-on common practices for managing biosamples; coordinating and maturing data, information standards, and strategies; and connecting to social benefit areas.

## The Omic BON Solution

The Group on Earth Observations Biodiversity Observation Network (GEO BON) endorsed the Omic Biodiversity Observation Network (Omic BON) to address the above challenges. Founding partners of Omic BON include observing networks, data/sample infrastructures, and standards and best practices organizations. Its overarching aim is to implement a meta-observatory of life at the molecular scale across earth systems (Box 1).

Omic BON will coordinate efforts along eight principal axes: (i) localized omic observatories; (ii) networks of observing platforms; (iii) data infrastructures; (iv) curated, long-term stores of biosamples; (v) (meta)data standardization bodies; (vi) coordination and integration with other biological and environmental observing; (vii) documentation and coordination practices and standards; and (viii) identification and iteration on requirements to benefit science, society, and nature.

Box 1: **Omic BON's vision, mission, and goals**

**Vision**

A sustainable, responsive, and globally integrated omic meta-observatory that monitors biodiversity at the molecular level.^1^

**Mission**

To transition the fragmented observations of biomolecular diversity into coordinated contributions to a meta-observatory for collective insight and action.

**Meta-observatory**

A distributed observatory to which anyone performing well-documented observations - from citizen science initiatives to established long-term observatories - can contribute. The observations conducted independently across time and space are integrated into a coordinated body of (meta)data through a harmonized community of practice, shared standards, and agreed-on methods. Benefits are shared among the contributors and with broader society for the common good.

**Goals**

- Provide a forum to discuss and coordinate omics methods, standards, and approaches among the land, ocean, freshwater, and human-health observing communities
- Facilitate standard protocol development to build reliable baselines of biomolecular diversity
- Facilitate calibration among partners as omic technologies develop, are adopted, and evolve, channeling innovations (new sequencing technologies, automated samplers, data science) into meta-observatory operations
- Support partners in overcoming regionalization and siloing of biomolecular observations, data, and applications
- Facilitate the establishment, sustainability, and interoperability of omic time series
- Facilitate sharing and sustained delivery of trusted biomolecular (meta)data and information products to global aggregators (International Nucleotide Sequence Database Collaboration [INSDC], Ocean Biodiversity Information System [OBIS], Global Biodiversity Information Facility [GBIF]), compatible with specifications relevant to the essential biodiversity variables (EBVs), essential ocean variables (EOVs), and other biodiversity monitoring mechanisms at local to global scales and with proper respect for ethical, legal, and social issues
- Highlight contributions of partners by establishing a regular global assessment of change in biomolecular diversity, reporting trends in biomolecular-based variables and indicators worldwide
- Support mechanisms to detect sudden or consequential events that facilitate appropriate authorities in considering collective, targeted actions in response to emerging threats (e.g., to health of humans, agriculture, aquaculture, and fisheries) or needs (e.g., in monitoring invasive species or illegal trade in protected species)

In order to achieve Omic BON's goals, we will start with three initial activities:

1. Establish an operational Omic BON with funding, governance, and administrative support
2. Develop Omic BON's data strategy to improve the availability, reusability, and interoperability of global omics biodiversity data and achieve the sustained delivery of key data/information products in support of the Omic BON goals
3. Establish Omic BON's strategy for tracking/indexing samples to build the meta-collection pillar of the meta-observatory, including recommended practices (in scientific, ethical, legal, social dimensions) for accessing, tracking, and storing the biosamples that underpin omic observations and future analyses of those samples (sharing samples, *ex situ* access)

These foundational activities will evolve and grow in number to reflect current and future priorities in omics observing. For up-to-date information on Omic BON activities, please consult our website [8].

^1^As such, Omic BON will address the finest scale of biodiversity, as noted in the CBD.

## Technical Description: Omic BON as a Social-Technological Infrastructure under GEO BON

### Where does Omic BON fit among global and regional programs?

Omic BON emerged through joint collaborations across existing initiatives. Omic BON was formed by the union of the Global Omics Observing Network (GLOMICON, an outcome of the AtlantOS project) and the Genomic Observatories Network (GOs Network [9], a collaboration of GEO BON and the Genomic Standards Consortium [GSC]). Forming Omic BON under GEO BON was envisioned in consultation with Marine BON (MBON) at the 2018 AtlantOS Workshop and the GSC21 meeting in 2019.

Omic BON was formally proposed to GEO BON in 2021 with the support of founding partners from

- Long-term observatories and observation networks: National Science Foundation's National Ecological Observatory Network (NEON), Marine Global Earth Observatory (MarineGEO), Agriculture and Agri-Food Canada (AAFC), European Marine Biological Resource Centre (EMBRC), Australian Microbiome Initiative (AM), and MBON
- Data and sample infrastructures: GBIF, OBIS, and Global Genome Biodiversity Network (GGBN)
- Standards and methodology management organizations: GSC, Biodiversity Information Standards (TDWG), and Ocean Best Practices System (OBPS)
- Global ocean observing networking programs: Global Ocean Observing System (GOOS) and Ocean Biomolecular Observing Network (OBON)

With the official endorsement in 2022, Omic BON became the first thematic BON focused on an observational technique.

Omic BON will continue to bring together observers and observatories across sectors and environments. We benefit from the coordination already occurring in the marine domain, in part due to the mobilization spurred by the United Nations Decade of Ocean Science for Sustainable Development (UN Ocean Decade), and similar but at present largely parallel efforts in the terrestrial, freshwater, atmospheric, and space/extra-terrestrial domain.

**Figure 1:**
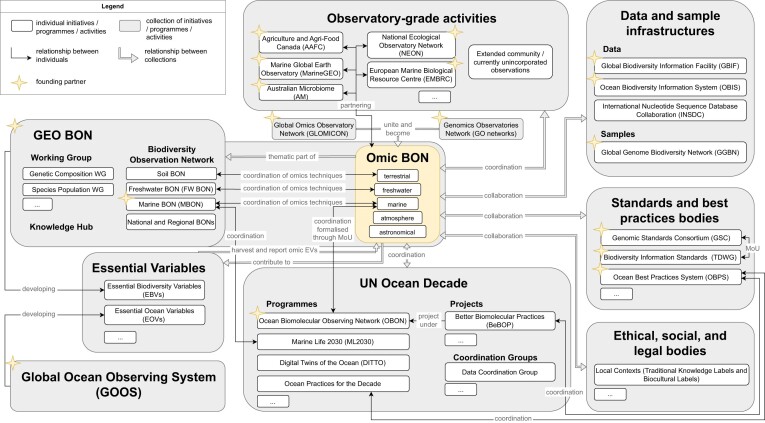
High-level positioning of Omic BON across related projects and initiatives. Omic BON originated out of the union of the Global Omics Observatory Network (GLOMICON) and the Genomics Observatories Network (GOs Network), partnering with established observatory-grade activities. With its partners, Omic BON will support the coordination of omics observations into a meta-observatory. Within GEO BON, Omic BON will complement thematic BONs focused on environments (Marine BON, Freshwater BON, Soil BON), as well as National and Regional BONs. Omic BON will additionally work closely with the GEO BON Genetic Composition Working Group as well as the Species Population Working Group and will coordinate with the relevant Knowledge Hubs as they arise. Further, Omic BON will contribute to the coordination of omics-enabled essential variables (EVs) between GEO BON and the Global Ocean Observing System (GOOS). Within the context of the UN Decade of Ocean Science for Sustainable Development, Omic BON will work closely with the Decade's Ocean Biomolecular Observing Network (OBON), which will be a key contributor to the marine component of Omic BON. Through OBON, Omic BON will further coordinate with the relevant Decade Actions such as the Marine Life 2030 (ML2030) program, the Digital Twins of the Ocean (DITTO) program, the Better Biomolecular Ocean Practices (BeBOP) project, and so on. Moreover, Omic BON will collaborate with the relevant data and sample infrastructures, such as the Global Biodiversity Information Facility (GBIF), the Ocean Biodiversity Information System (OBIS), the International Nucleotide Sequence Database Collaboration (INSDC), and the Global Genome Biodiversity Network (GGBN), with relevant standards and best practices bodies, such as the Genomic Standards Consortium (GSC), Biodiversity Information Standards (TDWG), and the Ocean Best Practices System (OBPS), to support their application and maturation, as well as with ethical, social and legal bodies, such as the Local Contexts initiative to ensure responsible practices.

Figure 1 shows the high-level positioning of Omic BON across related projects and initiatives. Organizations are encouraged to join Omic BON from across the omics biodiversity observing community to facilitate global collaboration and operationalization. The co-authors of this charter agree to champion Omic BON and help formalize the relationship between Omic BON and their respective organizations as necessary and appropriate.

### Governance and membership

**Figure 2:**
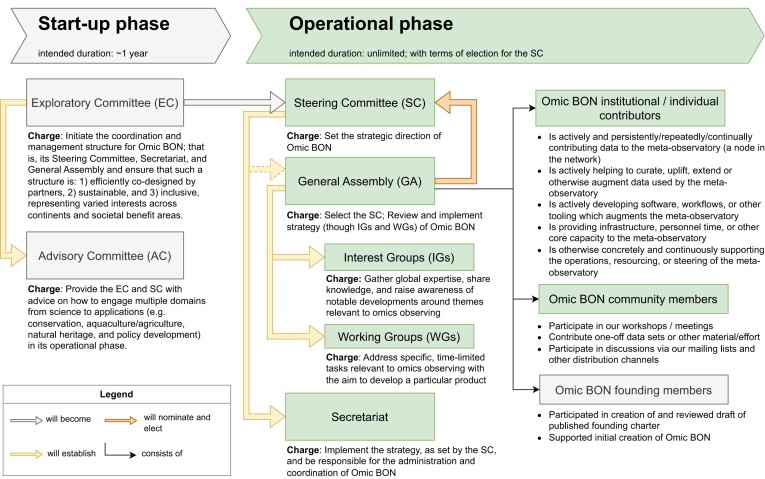
Omic BON's proposed governance structure and its parts main responsibilities. Start-up phase: Omic BON will formalize its organization during the start-up phase (anticipated duration 1 year) with 2 transitional committees: an *Exploratory Committee (EC)* tasked with coordination and management and an *Advisory Committee (AC)* representing key partner organizations. Operational phase: This will progress into the operational phase, where the initial committees will transition into clearly scoped bodies and mechanisms, operating along publicly released terms of reference. To transition, the EC will appoint the first *Steering Committee (SC)*, which will set the strategic direction of Omic BON. The SC will initiate the *Omic BON membership* and form a *General Assembly (GA)* thereof. In addition to the *founding members*, we envision the GA will include both *contributors* who are actively contributing or managing data as a node in the network and *community members* who participate in discussions and contribute in other ways toward the BON. Subsequently, members of the SC will be nominated and elected by the GA. Implementation or fulfillment of SC strategy and decisions will be carried out by a *Secretariat*, which is appointed by and reports to the SC. *Interest groups (IGs)* and *working groups (WGs)* will be formed based on the strategy laid out by the SC, which will put out a call for IGs and WGs every year at the annual meeting. Based on that call, motions to form IGs/WGs can be proposed by members of the GA and reviewed by the secretariat and SC.

To build a structural foundation for Omic BON's long-term success, it will operate with a defined governance structure with distributed responsibilities and terms. The initial governance structure of Omic BON is illustrated in Figure 2.

## Responsibilities in Omic Biodiversity Observation

Ethical, legal, and social considerations are essential in omics biodiversity observation to ensure responsible and sustainable practices. An example of particular importance for omics is the Nagoya Protocol of the CBD, which aims to ensure fair and equitable distribution of benefits derived from the study and utilization of genetic resources [10]. Recent developments consider how ABS provisions might extend to Digital Sequence Information (DSI) and, under the Law of the Sea, to areas of biodiversity beyond national jurisdiction (BBNJ). More broadly, Omic BON will address how to implement the CARE principles (e.g., implementation through the Traditional Knowledge and Biocultural Labels and Notices, see [11]). These mechanisms support Omic BON in making Indigenous data visible and transparent for Indigenous authority and governance. Additionally, it will be essential to develop a diversity, inclusion, and equity strategy to ensure that the interests and operational realities in Omic BON's scope are well represented. Further, the effective communication of research findings will be instrumental in enhancing public understanding and participation in biodiversity conservation. To achieve this, it is crucial to ensure quick dissemination of trusted information products, along with realistic and transparent information about the capabilities and limitations of omics, including environmental DNA, technologies. Collaboration between researchers, policymakers, private sector, and other stakeholders is key to developing guidelines and pipelines that ensure responsible, inclusive, and effective/informative practices in omics biodiversity observation. With these aspects, omics biodiversity observation can build a foundation to uphold ethical standards, comply with legal frameworks, and promote positive social outcomes.

## Conclusions

Omic BON will serve the global omics biodiversity community through open, trusted, and inclusive coordination. This is crucial to help coordinate omics research and technology information to effectively and sustainably contribute to the global baselines and trusted indicators needed to address pressing threats to the biosphere and opportunities for conservation and sustainable development. We envisage that the Omic BON community will establish a meta-observatory with decadal strategies and interoperability models, forging sustained links to an ever-growing collection of stakeholders and global programs. This fundamental step in mainstreaming omic approaches will help build the collective capabilities and intelligence needed to address the grand scientific and societal challenges of our time.

## Supplementary Material

giad068_GIGA-D-23-00062_Original_Submission

giad068_GIGA-D-23-00062_Revision_1

giad068_Response_to_Reviewer_Comments_Original_Submission

giad068_Reviewer_1_Report_Original_Submission Melody Clark -- 7/18/2023 Reviewed

## Data Availability

Not applicable

## Abbreviations

AAFC: Agriculture and Agri-Food Canada; ABS: Access and Benefit Sharing; AC: Advisory Committee; AM: Australian Microbiome Initiative; AtlantOS project: All-Atlantic Ocean Observing System, EU H2020 project; BBNJ: Biodiversity Beyond National Jurisdiction; BC Labels and Notices: Biocultural Labels and Notices; BeBOP: Better Biomolecular Ocean Practices; BON: Biodiversity Observation Network; CARE: Collective Benefit—Authority to Control—Responsibility—Ethics; CBD: Convention on Biological Diversity; DITTO: Digital Twins of the Oceans; DSI: Digital Sequence Information; EBV: essential biodiversity variable; EC: Exploratory Committee; EOV: essential ocean variable; FAIR: Findable, Accessible, Interoperable, Reusable; GA: General Assembly; GBIF: Global Biodiversity Information Facility; GEO BON: Group on Earth Observations Biodiversity Observation Network; GLOMICON: Global Omics Observatory Network; GO Network: Genomics Observatories Network; GOOS: Global Ocean Observing System; GSC: Genomic Standards Consortium; IG: Interest Group; INSDC: International Nucleotide Sequence Database Collaboration; ML2030: Marine Life 2030; NEON: National Ecological Observatory Network; OBIS: Ocean Biodiversity Information System; OBON: Ocean Biomolecular Observing Network; OBPS: Ocean Best Practices System; Omic BON: Omic Biodiversity Observation Network; SC: Steering Committee; TDWG: Biodiversity Information Standards; TK Labels and Notices: Traditional Knowledge Labels and Notices; UN Ocean Decade: United Nations Decade of Ocean Science for Sustainable Development; UN Decade on Restoration: United Nations Decade on Ecosystem Restoration; WG: Working Group.

## References

[1] Bourlat SJ, Borja A, Glibert J, et al. Genomics in marine monitoring: new opportunities for assessing marine health status. Mar Pollut Bull. 2013;74:19–31.. 10.1016/j.marpolbul.2013.05.042. 23806673

[2] Beale DJ, Jones OAH, Bose U, et al. Omics-based ecosurveillance for the assessment of ecosystem function, health, and resilience. Emerg Top Life Sci. 2022;6:185–99.. 10.1042/ETLS20210261. 35403668 PMC9023019

[3] Díaz S, Settle J, Brondízio E. Summary for policymakers of the global assessment report on biodiversity and ecosystem services of the Intergovernmental Science-Policy Platform on Biodiversity and Ecosystem Services. 2019. IPBES Secretariat. *Zenodo*. 10.5281/zenodo.3553579.

[4] Halpern BS, Longo C, Hardy D, et al. An index to assess the health and benefits of the global ocean. Nature. 2012;488:615–20.. 10.1038/nature11397. 22895186

[5] Wilkinson MD, Dumontier M, Aalbersberg IJJ, et al. The FAIR Guiding Principles for scientific data management and stewardship. Sci Data. 2016;3:160018. 10.1038/sdata.2016.18. 26978244 PMC4792175

[6] Carroll SR, Garba I, Figueroa-Rodríguez OL, et al. The CARE Principles for indigenous data governance. Data Sci J. 2020;19:43. 10.5334/dsj-2020-043.

[7] Aubertin C, Nivart A. Nature in Common: Beyond the Nagoya Protocol. Marseille: IRD Éditions, 2021.

[8] Omic BON . https://geobon.org/bons/thematic-bon/omic-bon/. Accessed 9 December 2022.

[9] Davies N, Field D, Amaral-Zettler L, et al. The founding charter of the Genomic Observatories Network. Gigascience 2014;3:2. 10.1186/2047-217X-3-2. PMC3995929 24606731

[10] Mc Cartney AM, Head MA, Tsosie KS, et al. Indigenous peoples and local communities as partners in the sequencing of global eukaryotic biodiversity. NPJ Biodivers. 2023;2:8. 10.1038/s44185-023-00013-7. 38693997 PMC11062294

[11] Local Contexts . https://localcontexts.org. Accessed 14 June 2023.

