# Supplementary material for: The founding charter of the Omic Biodiversity Observation Network (Omic BON)
Source: Gigascience. 2023 Aug 26;12:giad068. doi: 10.1093/gigascience/giad068 (PMC10460158; doi:10.1093/gigascience/giad068)
Supplement: giad068_GIGA-D-23-00062_Revision_1 [file giad068_giga-d-23-00062_revision_1.pdf]

GigaScience

The founding charter of the Omic Biodiversity Observation Network (Omic BON)

--Manuscript Draft--

**Manuscript Number:**	GIGA-D-23-00062R1
**Full Title:**	The founding charter of the Omic Biodiversity Observation Network (Omic BON)
**Article Type:**	Commentary
**Funding Information:**	
**Abstract:**	Omic BON is a thematic Biodiversity Observation Network under the Group on Earth Observations Biodiversity Observation Network (GEO BON), focused on coordinating the observation of biomolecules in organisms and the environment. Our founding partners include representatives from national, regional, and global observing systems, standards organizations, and data and sample management infrastructures. By coordinating observing strategies, methods, and data flows, Omic BON will facilitate the co-creation of a global omics meta-observatory to generate actionable knowledge. Here, we present key elements of Omic BON's founding charter and first activities.
**Corresponding Author:**	Raïssa Meyer<br>Alfred Wegener Institute for Polar and Marine Research<br>Bremerhaven, GERMANY
**Corresponding Author Secondary Information:**	
**Corresponding Author's Institution:**	Alfred Wegener Institute for Polar and Marine Research
**Corresponding Author's Secondary Institution:**	
**First Author:**	Raïssa Meyer
**First Author Secondary Information:**	
**Order of Authors:**	Raïssa Meyer
	Neil Davies
	Kathleen J Pitz
	Chris Meyer
	Robyn Samuel
	Jane Hudson
	Ward Appeltans
	Katharine Barker
	Francisco Chavez
	J. Emmett Duffy
	Kelly D. Goodwin
	Maui Hudson
	Margaret E. Hunter
	Johannes Karstensen
	Christine Laney
	Margaret Leinen
	Paula Mabee
	James A. Macklin
	Frank Muller-Karger
	Nicolas Pade

Powered by Editorial Manager® and ProduXion Manager® from Aries Systems Corporation

	Jay Pearlman
	Lori Phillips
	Pieter Provoost
	Ioulia Santi
	Dmitry Schigel
	Lynn M. Schriml
	Alice Soccodato
	Saara Suominen
	Kate Thibault
	Visotheary Ung
	Jodie van de Kamp
	Elycia Wallis
	Ramona Walls
	Pier Luigi Buttigieg
**Order of Authors Secondary Information:**	
**Response to Reviewers:**	Dear Nicole Nogoy and Melody Clark,<br><br>Thank you for your positive response to our manuscript "The founding charter of the Omic Biodiversity Observation Network (Omic BON)".<br><br>I am thrilled to hear that you are considering it for publication in GigaScience and have made the requested formatting revisions to the references. Please let me know if any further changes are required.<br><br>I am delighted to hereby submit the revised version of the manuscript.<br><br>With thanks and regards,<br>Raïssa Meyer
**Additional Information:**	
**Question**	**Response**
Are you submitting this manuscript to a special series or article collection?	No
**Experimental design and statistics**<br><br>Full details of the experimental design and statistical methods used should be given in the Methods section, as detailed in our Minimum Standards Reporting Checklist. Information essential to interpreting the data presented should be made available in the figure legends.<br><br>Have you included all the information requested in your manuscript?	No

Powered by Editorial Manager® and ProduXion Manager® from Aries Systems Corporation

If not, please give reasons for any omissions below.<br><br>as follow-up to "**Experimental design and statistics**<br><br>Full details of the experimental design and statistical methods used should be given in the Methods section, as detailed in our Minimum Standards Reporting Checklist. Information essential to interpreting the data presented should be made available in the figure legends.<br><br>Have you included all the information requested in your manuscript?<br><br>"	Not applicable
**Resources**<br><br>A description of all resources used, including antibodies, cell lines, animals and software tools, with enough information to allow them to be uniquely identified, should be included in the Methods section. Authors are strongly encouraged to cite Research Resource Identifiers (RRIDs) for antibodies, model organisms and tools, where possible.<br><br>Have you included the information requested as detailed in our Minimum Standards Reporting Checklist?	No
If not, please give reasons for any omissions below.<br><br>as follow-up to "**Resources**<br><br>A description of all resources used, including antibodies, cell lines, animals and software tools, with enough information to allow them to be uniquely identified, should be included in the	Not applicable

Methods section. Authors are strongly encouraged to cite Research Resource Identifiers (RRIDs) for antibodies, model organisms and tools, where possible.<br><br>Have you included the information requested as detailed in our Minimum Standards Reporting Checklist?<br><br>"	
**Availability of data and materials**<br><br>All datasets and code on which the conclusions of the paper rely must be either included in your submission or deposited in publicly available repositories (where available and ethically appropriate), referencing such data using a unique identifier in the references and in the “Availability of Data and Materials” section of your manuscript.<br><br>Have you have met the above requirement as detailed in our Minimum Standards Reporting Checklist?	No
If not, please give reasons for any omissions below.<br><br>as follow-up to "**Availability of data and materials**<br><br>All datasets and code on which the conclusions of the paper rely must be either included in your submission or deposited in publicly available repositories (where available and ethically appropriate), referencing such data using a unique identifier in the references and in the “Availability of Data and Materials” section of your manuscript.<br><br>Have you have met the above requirement as detailed in our Minimum Standards Reporting Checklist?	Not applicable

"	

Powered by Editorial Manager® and ProduXion Manager® from Aries Systems Corporation

Manuscript

Click here to access/download;Manuscript;RMeyer et al_Omic BON founding charter.docx

The founding charter of the Omic Biodiversity Observation Network (Omic BON)

Raïssa Meyer [1,2,3 * +], Neil Davies [4,5 * +], Kathleen J Pitz [6 +], Chris Meyer [7 +], Robyn Samuel [8,9 +], Jane Anderson [10 #], Ward Appeltans [11], Katharine Barker [12], Francisco Chavez [6], J. Emmett Duffy [13], Kelly D. Goodwin [14 #], Maui Hudson [15 #], Margaret E. Hunter [16], Johannes Karstensen [17], Christine Laney [18 #], Margaret Leinen [19], Paula Mabee [18 #], James A. Macklin [20 #], Frank Muller-Karger [21], Nicolas Pade [22 #], Jay Pearlman [23], Lori Phillips [24 #], Pieter Provoost [11], Ioulia Santi [22,25], Dmitry Schigel [26], Lynn M. Schriml [27 #], Alice Soccodato [22], Saara Suominen [11 #], Katherine M. Thibault [18 #], Visotheary Ung [28 #], Jodie van de Kamp [29 #], Elycia Wallis [20 #], Ramona Walls [31 #], Pier Luigi Buttigieg [1,32 +]

* corresponding authors

Raïssa Meyer, raissa.meyer@awi.de; Neil Davies, ndavies@moorea.berkeley.edu

+ Omic BON Exploratory Committee

Omic BON Advisory Committee

1 Alfred Wegener Institute, Helmholtz Centre for Polar and Marine Research, Am Handelshafen 12, 27570 Bremerhaven, Germany; ORCID: 0000-0002-2996-719X; email: raissa.meyer@awi.de

2 Faculty of Geosciences, University of Bremen, Klagenfurter Str. 2-4, 28359 Bremen, Germany

3 Max Planck Institute for Marine Microbiology, Celsiusstrasse 1, 28359 Bremen, Germany

4 Gump South Pacific Research Station, University of California Berkeley, BP 244, Maharepa - 98728, Moorea, French Polynesia; ORCID: 0000-0001-8085-5014; email: ndavies@moorea.berkeley.edu

5 Berkeley Institute for Data Science, University of California, Berkeley, CA 94720, USA

6 Monterey Bay Aquarium Research Institute, 7700 Sandholdt Road, Moss Landing, 95039 CA, United States; ORCID: 0000-0002-4931-8592; email: kpitz@mbari.org

7 Department of Invertebrate Zoology, National Museum of Natural History, Smithsonian Institution, 10th and Constitution Ave. NW, Washington, 20560 D.C., United States; ORCID: 0000-0001-8085-5014; email: meyerc@si.edu

8 School of Ocean and Earth Science, University of Southampton, University Road, Southampton, SO17 1BJ United Kingdom; ORCID: 0000-0001-5989-4588; email: R.M.Samuel@soton.ac.uk

9 Ocean Technology and Engineering Group, National Oceanography Center, European Way, Southampton, SO14 3ZH, United Kingdom

10 New York University, 50 West 4th Street, New York City, 10012 NY, United States, ORCID: 0000-0002-3304-0477, ja77@nyu.edu

11 Intergovernmental Oceanographic Commission of UNESCO, Ocean Biodiversity Information System, Jacobsenstraat 1, 8400 Oostende, Begium; ORCID: 0000-0002-3237-4547; email: w.appeltans@unesco.org;

12 Global Genome Biodiversity Network Secretariat Office, National Museum of Natural History, Smithsonian Institution, 10th and Constitution Ave. NW, Washington, 20560 D.C., United States; ORCID: 0000-0002-4788-0223; barkerk@si.edu

6 Monterey Bay Aquarium Research Institute, 7700 Sandholdt Road, Moss Landing, 95039 CA, United States; ORCID: 0000-0002-0691-292x; email: chfr@mbari.org

13 Tennenbaum Marine Observatories Network and MarineGEO program, Smithsonian Environmental Research Center, 647 Contees Wharf Road, Edgewater, 21037 MD, United States; ORCID: 0000-0001-8595-6391; email: duffye@si.edu

14 National Oceanic & Atmospheric Administration, NOAA Ocean Exploration, 8901 La Jolla Shores Dr., La Jolla, 92037 CA, United States; ORCID: 0000-0001-9583-8073; email: kelly.goodwin@noaa.gov

15 Te Kotahi Research Institute, University of Waikato, Knighton Rd Hamilton 3240, New Zealand, ORCID: 0000-0003-3880-4015, maui.hudson@waikato.ac.nz

16 U.S. Geological Survey, Wetland and Aquatic Research Center, 7920 NW 71st Street, Gainesville, 32653 Florida, United States; ORCID: 0000-0002-4760-9302; email: mhunter@usgs.gov

17 GEOMAR Helmholtz Centre for Ocean Research Kiel, Duesternbrooker Weg 20, 24105 Kiel, Germany; ORCID: 0000-0001-5044-7079; email: jkarstensen@geomar.de

18 National Ecological Observatory Network, Battelle, 1685 38th St #100, Boulder, 80301 Colorado, United States; ORCID: 0000-0002-4944-2083; email: claney@battelleecology.org

19 Scripps Institution of Oceanography, 9500 Gilman Drive, La Jolla, 92093 CA, United States; ORCID: 0000-0002-7982-3661; email: mleinen@ucsd.edu

18 National Ecological Observatory Network, Battelle, 1685 38th St #100, Boulder, 80301 Colorado, United States; ORCID: 0000-0002-8455-3213; email: mabee@battelleecology.org

20 Agriculture and Agri-Food Canada (AAFC), 960 Carling Avenue, K1A 0C6, Ontario, Canada; ORCID: 0000-0001-9508-1349; email: james.macklin@agr.gc.ca

21 University of South Florida, College of Marine Science, 140 7th Ave. South, St. Petersburg, 33701 Florida, United States; ORCID: 0000-0003-3159-5011; email: carib@usf.edu

22 European Marine Biological Resource Centre (EMBRC-ERIC), 4 Place Jussieu, 75252 Paris, France; ORCID: 0000-0003-2733-9752; email: nicolas.pade@embrc.eu

23 IEEE, 11 rue de l'Amiral Hamelin, 75116 Paris, France; email: jay.pearlman@ieee.org

24 Agriculture and Agri-Food Canada (AAFC), 2585 County Road 20, Harrow, N0R 1G0, Ontario; ORCID: 0000-0003-1005-5331; email: lori.phillips@agr.gc.ca

11 Intergovernmental Oceanographic Commission of UNESCO, Ocean Biodiversity Information System, Jacobsenstraat 1, 8400 Oostende, Begium; ORCID: 0000-0002-4236-0384; email: p.provoost@unesco.org

22 European Marine Biological Resource Centre (EMBRC-ERIC), 4 Place Jussieu, 75252 Paris, France; ORCID: 0000-0002-0202-8256; email: ioulia.santi@embrc.eu

25 Hellenic Centre for Marine Research (HCMR), Institute of Marine Biology, Biotechnology and Aquaculture (IMBBC), P.O. Box 2214, Heraklion, GR71003, Greece

26 GBIF | Global Biodiversity Information Facility, Secretariat, Universitetsparken 15, Copenhagen Ø, DK-2100, Denmark; ORCID: 0000-0002-2919-1168; email: dschigel@gbif.org

27 University of Maryland School of Medicine, 670 W. Baltimore St., Baltimore, MD 21201, United States; ORCID: 0000-0001-8910-9851; email: lschriml@som.umaryland.edu

22 European Marine Biological Resource Centre (EMBRC-ERIC), 4 Place Jussieu, 75252 Paris, France; ORCID: 0000-0003-4321-9312; email: alice.soccodato@embrc.eu

11 Intergovernmental Oceanographic Commission of UNESCO, Ocean Biodiversity Information System, Jacobsenstraat 1, 8400 Oostende, Begium; ORCID: 0000-0001-9401-8460; email: s.suominen@unesco.org

18 National Ecological Observatory Network, Battelle, 1685 38th St #100, Boulder, 80301 Colorado, United States; ORCID: 0000-0003-3477-6424; email: kthibault@battelleecology.org

28 CNRS-MNHN-SU-EPHE-UA, CP39, 18 ISYEB, Rue Buffon, 75005 Paris, France; ORCID: 0000-0002-4049-0820; email: visotheary.ung@mnhn.fr

29 CSIRO Environment, Castray Esplanade, Hobart, 7004 Tasmania, Australia; ORCID: 0000-0003-2167-0938, email: jodie.vandekamp@csiro.au

30 CSIRO, Melbourne, 3071 Australia; ORCID: 0000-0001-6017-0894; email: Ely.Wallis@csiro.au

31 Critical Path Institute, 1840 E River Road, Tucson, 85718 AZ, United States; ORCID: 0000-0001-8815-0078; email: rwalls@c-path.org

1 Alfred Wegener Institute, Helmholtz Centre for Polar and Marine Research, Am Handelshafen 12, 27570 Bremerhaven, Germany; ORCID: 0000-0002-4366-3088; email: pier.buttigieg@awi.de

32 Helmholtz Metadata Collaboration / GEOMAR Helmholtz Centre for Ocean Research Kiel, Duesternbrooker Weg 20, 24105 Kiel, Germany

Keywords

Biodiversity, Omics, eDNA, Earth Observation, GEO BON, Essential Variables, Biomonitoring

Abstract

Omic BON is a thematic Biodiversity Observation Network under the Group on Earth Observations Biodiversity Observation Network (GEO BON), focused on coordinating the observation of biomolecules in organisms and the environment. Our founding partners include representatives from national, regional, and global observing systems, standards organizations, and data and sample management infrastructures. By coordinating observing strategies, methods, and data flows, Omic BON will facilitate the co-creation of a global omics meta-observatory to generate actionable knowledge. Here, we present key elements of Omic BON's founding charter and first activities.

Background

Omics biodiversity observation - challenge and opportunity

All life on earth - from microbes to giant sequoias - depends on biomolecules involved in the transfer of information, such as genes, transcripts, and proteins. Analysis of these biomolecules can help us monitor biodiversity and understand how it is changing in response to human activities, environmental or biological pressures, or due to genetic drift or mutations. Molecular techniques, collectively known as *omics*, offer a powerful toolset for such investigations.

Referring to the holistic study of elements that compose a greater whole, *omics* is used by the biomolecular community to describe the study of DNA and RNA sequences, proteins, metabolites, and other biomolecules in (I) organisms (genomics, transcriptomics, proteomics, or metabolomics), or in (II) environmental material samples (eDNA/RNA, metagenomics, metatranscriptomics, metaproteomics, and metabolomics analyses).

Widely applied in biomedical research, omics has great potential in environmental and biodiversity studies [1,2]. Various socio-technical challenges, however, continue to impede the use of biomolecular evidence in public policy and resource management options [3,4]. The challenges to be surmounted include: (I) insufficient coordination among the various actors involved in biodiversity omics, (II) sparse and infrequent omic observations across many regions, environments, or ecosystems, especially with respect to baseline and time-series data, (III) insufficient convention and agreement on common practices for accessing, tracking, and storing the biosamples that underpin omic analyses, and (IV) a lack of standardized practices and operationalization of FAIR (Findable, Accessible, Interoperable, Reusable) [5] and CARE (Collective Benefit, Authority to Control, Responsibility, and Ethics) [6] data principles. Taken together, these obstacles challenge the seamless integration of omic observations in scientific syntheses, and can have significant ethical, legal and social implications (e.g., contributing to the limited success of Convention on Biological Diversity (CBD) Access & Benefit Sharing (ABS) provisions [7]).

Addressing these challenges will require global coordination of key actors; overcoming regional silos by ensuring compatibility of baselines, time-series, and reference libraries; developing concerted strategies and agreed common practices for managing biosamples; coordinating and maturing data, information standards, and strategies; and connecting to social benefit areas.

Main text

The Omic BON Solution

The GEO BON endorsed the Omic Biodiversity Observation Network (Omic BON) to address the above challenges. Founding partners of Omic BON include observing networks, data/sample infrastructures, and standards and best practices organizations. Its overarching aim is to implement a meta-observatory of life at the molecular scale across earth systems (Box 1).

Omic BON will coordinate efforts along eight principal axes: (I) localized omic observatories, (II) networks of observing platforms, (III) data infrastructures, (IV) curated, long-term stores of biosamples, (V) (meta)data standardization bodies, (VI) coordinating and integrating with other biological and environmental observing, (VII) documenting and coordinating practices and standards and (VIII) identifying and iterating on requirements to benefit science, society, and nature.

BOX 1.

Vision

A sustainable, responsive, and globally integrated omic meta-observatory that monitors biodiversity at the molecular level[1].

Mission

[1] As such, Omic BON will address the finest scale of biodiversity, as noted in the CBD

To transition the fragmented observations of biomolecular diversity into coordinated contributions to a meta-observatory for collective insight and action.

Meta-observatory

A distributed observatory to which anyone performing well-documented observations - from citizen science initiatives to established long-term observatories - can contribute. The observations conducted independently across time and space are integrated into a coordinated body of (meta)data through a harmonized community of practice, shared standards, and agreed methods. Benefits are shared among the contributors and with broader society for the common good.

Goals

- Provide a forum to discuss and coordinate omics methods, standards, and approaches among the land-, ocean-, freshwater, and human-health observing communities
- Facilitate standard protocol development to build reliable baselines of biomolecular diversity
- Facilitate calibration among partners as omic technologies develop, are adopted, and evolve, channeling innovations (new sequencing technologies, automated samplers, data science) into meta-observatory operations
- Support partners in overcoming regionalisation and siloing of biomolecular observations, data, and applications
- Facilitate the establishment, sustainability, and interoperability of omic time-series
- Facilitate sharing and sustained delivery of trusted biomolecular (meta)data and information products to global aggregators (International Nucleotide Sequence

Database Collaboration [INSDC], Ocean Biodiversity Information System [OBIS], Global Biodiversity Information Facility [GBIF]), compatible with specifications relevant to the Essential Biodiversity Variables (EBVs), Essential Ocean Variables (EOVs), and other biodiversity monitoring mechanisms at local to global scales, and with proper respect for ethical legal and social issues

- Highlight contributions of partners by establishing a regular global assessment of change in biomolecular diversity, reporting trends in biomolecular-based variables and indicators worldwide
- Support mechanisms to detect sudden or consequential events that facilitate appropriate authorities in considering collective, targeted actions in response to emerging threats (e.g., to health of humans, agriculture, aquaculture, and fisheries) or needs (e.g., in monitoring invasive species or illegal trade in protected species)

In order to achieve Omic BON's goals, we will start with three initial activities:

1. Establish an operational Omic BON with funding, governance, and administrative support
2. Develop Omic BON's Data Strategy to improve the availability, re-usability, and interoperability of global omics biodiversity data and achieve the sustained delivery of key data/information products in support of the Omic BON goals
3. Establish Omic BON's strategy for tracking/indexing samples to build the meta-collection pillar of the meta-observatory, including recommended practices (in scientific, ethical, legal, social dimensions) for accessing, tracking, and storing the biosamples that underpin omic observations and future analyses of those samples (sharing samples, ex-situ access)

These foundational activities will evolve and grow in number to reflect current and future priorities in omics observing. For up-to-date information on Omic BON activities, please consult our website [8].

Technical Description: Omic BON as a social-technological infrastructure under GEO BON

Where does Omic BON fit among global and regional programmes?

Omic BON emerged through joint collaborations across existing initiatives. Omic BON was formed by the union of the Global Omics Observing Network (GLOMICON, an outcome of AtlantOS project) and the Genomic Observatories Network (GOs Network [9], a collaboration of GEO BON and the Genomic Standards Consortium [GSC]). Forming Omic BON under GEO BON was envisioned in consultation with Marine BON (MBON) at the 2018 AtlantOS Workshop and the GSC21 meeting in 2019.

Omic BON was formally proposed to GEO BON in 2021 with the support of founding partners from

- long-term observatories and observation networks: National Science Foundation's National Ecological Observatory Network (NEON), Marine Global Earth Observatory (MarineGEO), Agriculture and Agri-Food Canada (AAFC), European Marine Biological Resource Centre (EMBRC), Australian Microbiome Initiative (AM), MBON,
- data and sample infrastructures: GBIF, OBIS, Global Genome Biodiversity Network (GGBN),

- standards and methodology management organizations: GSC, Biodiversity Information Standards (TDWG), Ocean Best Practices System (OBPS), and
- global ocean observing networking programmes: Global Ocean Observing System (GOOS), Ocean Biomolecular Observing Network (OBON).

With the official endorsement in 2022, Omic BON became the first thematic BON focused on an observational technique.

Omic BON will continue to bring together observers and observatories across sectors and environments. We benefit from the coordination already occurring in the marine domain, in part due to the mobilization spurred by the United Nations Decade of Ocean Science for Sustainable Development (UN Ocean Decade), and similar but at present largely parallel efforts in the terrestrial, freshwater, atmospheric, and space/extra-terrestrial domain.

Figure 1 shows the high level positioning of Omic BON across related projects and initiatives. Organizations are encouraged to join Omic BON from across the omics biodiversity observing community to facilitate global collaboration and operationalization. The co-authors of this charter agree to champion Omic BON and help formalize the relationship between Omic BON and their respective organizations as necessary and appropriate.

Figure 1: *High level positioning of Omic BON across related projects and initiatives.* Omic BON originated out of the union of the Global Omics Observatory Network (GLOMICON) and the Genomics Observatories Network (GOs Network), partnering with established observatory-grade activities. With its partners, Omic BON will support the coordination of omics observations into a meta-observatory. Within GEO BON, Omic BON will complement thematic BONs focused on environments (Marine BON, Freshwater BON, Soil BON), as well as National and Regional BONs. Omic BON will additionally work closely with the GEO BON Genetic Composition Working Group as well as the Species Population Working Group, and will coordinate with the relevant Knowledge Hubs as they arise. Further, Omic BON will contribute to the coordination of omics-enabled Essential Variables (EVs) between

GEO BON and the Global Ocean Observing System (GOOS). Within the context of the UN Decade of Ocean Science for Sustainable Development, Omic BON will work closely with the Decade's Ocean Biomolecular Observing Network (OBON), which will be a key contributor to the marine component of Omic BON. Through OBON, Omic BON will further coordinate with the relevant Decade Actions such as the Marine Life 2030 (ML2030) Programme, the Digital Twins of the Ocean (DITTO) Programme, the Better Biomolecular Ocean Practices (BeBOP) Project, etc. Moreover, Omic BON will collaborate with the relevant data and sample infrastructures, such as the Global Biodiversity Information Facility (GBIF), the Ocean Biodiversity Information System (OBIS), the International Nucleotide Sequence Database Collaboration (INSDC), and the Global Genome Biodiversity Network (GGBN), with relevant standards and best practices bodies, such as the Genomic Standards Consortium (GSC), Biodiversity Information Standards (TDWG), and the Ocean Best Practices System (OBPS) to support their application and maturation, as well as with ethical, social and legal bodies, such as the Local Contexts initiative to ensure responsible practices.

Governance and membership

To build a structural foundation for Omic BON's long-term success, it will operate with a defined governance structure with distributed responsibilities and terms. The initial governance structure of Omic BON is illustrated in Figure 2.

Figure 2: *Omic BON's proposed governance structure and its parts main responsibilities.*

Start-up Phase: Omic BON will formalize its organization during the Start-up Phase (anticipated duration 1 year) with two transitional committees: an *Exploratory Committee (EC)* tasked with coordination and management and an *Advisory Committee (AC)* representing key partner organizations.

Operational Phase: This will progress into the operational phase, where the initial committees will transition into clearly scoped bodies and mechanisms, operating along publicly released terms of reference. To transition, the EC will appoint the first *Steering Committee (SC)* which will set the strategic direction of Omic BON. The SC will initiate the *Omic BON membership* and form a *General Assembly (GA)* thereof. In addition to the *founding members*, we envision the GA will include both *contributors* who are actively contributing or managing data as a node in the network and *community members* who participate in discussions and contribute in other ways towards the BON. Subsequently, members of the SC will be nominated by and elected by the GA. Implementation or fulfillment of SC strategy and decisions will be carried out by a *Secretariat*, which is appointed by and reports to the SC. *Interest groups (IGs)* and *working groups (WGs)* will be formed based on the strategy laid out by the SC, which will put out

a call for IGs and WGs every year at the annual meeting. Based on that call, motions to form IGs/WGs can be proposed by members of the GA, and reviewed by the secretariat and SC.

Responsibilities in omic biodiversity observation

Ethical, legal, and social considerations are essential in omics biodiversity observation to ensure responsible and sustainable practices. An example of particular importance for omics is the Nagoya Protocol of the CBD, which aims to ensure fair and equitable distribution of benefits derived from the study and utilization of genetic resources [10]. Recent developments consider how ABS provisions might extend to Digital Sequence Information (DSI), and under the Law of the Sea, to areas of biodiversity beyond national jurisdiction (BBNJ). More broadly, Omic BON will address how to implement the CARE principles (e.g., implementation through the Traditional Knowledge and Biocultural Labels and Notices - see https://localcontexts.org). These mechanisms support Omic BON in making Indigenous data visible and transparent for Indigenous authority and governance. Additionally, it will be essential to develop a diversity, inclusion, and equity strategy to ensure that the interests and operational realities in Omic BON’s scope are well represented. Further, the effective communication of research findings will be instrumental in enhancing public understanding and participation in biodiversity conservation. To achieve this, it is crucial to ensure quick dissemination of trusted information products, along with realistic and transparent information about the capabilities and limitations of omics, including eDNA, technologies. Collaboration between researchers, policymakers, private sector, and other stakeholders is key to developing guidelines and pipelines that ensure responsible, inclusive, and effective/informative practices in omics biodiversity observation. With these aspects, omics biodiversity observation can build a foundation to uphold ethical standards, comply with legal frameworks, and promote positive social outcomes.

Conclusions

The Omic BON will serve the global omics biodiversity community through open, trusted and inclusive coordination. This is crucial to help coordinate omics research and technology information to effectively and sustainably contribute to the global baselines and trusted indicators needed to address pressing threats to the biosphere and opportunities for conservation and sustainable development. We envisage that the Omic BON community will establish a meta-observatory with decadal strategies and interoperability models, forging sustained links to an ever-growing collection of stakeholders and global programmes. This fundamental step in mainstreaming omic approaches will help build the collective capabilities and intelligence needed to address grand scientific and societal challenges of our time.

Table 1: List of abbreviations (in alphabetical order).

AAFC	Agriculture and Agri-Food Canada
ABS	Access and Benefit Sharing
AC	Advisory Committee
AM	Australian Microbiome
AtlantOS project	All-Atlantic Ocean Observing System, EU H2020 project
BBNJ	Biodiversity Beyond National Jurisdiction
BC Labels and Notices	Biocultural Labels and Notices
BeBOP	Better Biomolecular Ocean Practices
BON	Biodiversity Observation Network
CARE principles	CARE (Collective Benefit - Authority to Control - Responsibility - Ethics) Principles for Indigenous Data Governance
CBD	Convention on Biological Diversity
DITTO	Digital Twins of the Oceans
DSI	Digital Sequence Information
EBV	Essential Biodiversity Variable
eDNA	environmental DNA
EOV	Essential Ocean Variable
EC	Exploratory Committee

FAIR data principles	FAIR (Findable - Accessible - Interoperable - Reusable) data principles
GBIF	Global Biodiversity Information Facility
GA	General Assembly
GEO BON	Group on Earth Observations Biodiversity Observation Network
GLOMICON	Global Omics Observatory Network
GO Network	Genomics Observatories Network
GOOS	Global Ocean Observing System
GSC	Genomic Standards Consortium
INSDC	International Nucleotide Sequence Database Collaboration
IG	Interest Group
ML2030	Marine Life 2030
NEON	National Ecological Observatory Network
OBIS	Ocean Biodiversity Information System
OBON	Ocean Biomolecular Observing Network
OBPS	Ocean Best Practices System
Omic BON	Omic Biodiversity Observation Network
SC	Steering Committee

TDWG	Biodiversity Information Standards (TDWG)
TK Labels and Notices	Traditional Knowledge Labels and Notices
UN Ocean Decade	United Nations Decade of Ocean Science for Sustainable Development
UN Decade on Restoration	United Nations Decade on Ecosystem Restoration
WG	Working Group

Data Availability

Not applicable

Declarations

Ethics approval and consent to participate

Not applicable

Consent for publication

Not applicable

Data Availability

Not applicable

Competing interests

The authors declare that they have no competing interests.

Funding

RM was supported by the European Union’s Horizon 2020 Research and Innovation Programmes under grant agreement N° 862923, project AtlantECO (Atlantic ECOsystem assessment, forecasting and sustainability), and grant agreement N° 862626, project EuroSea (Improving and Integrating European Ocean Observing and Forecasting Systems for Sustainable use of the Oceans). ND and CM were supported by the U.S. National Science Foundation (NSF) awards: N° 2129268 and N° 2004642; any opinions, findings, and conclusions or recommendations expressed in this material are those of the authors and do not necessarily reflect the views of the NSF. KJP was supported by the David and Lucile Packard Foundation. PLB was supported through the Helmholtz Metadata Collaboration. RS was supported by the Natural Environment Research Council (NERC) NEXUSS Studentship grant N° NE/N012070/1. CL, KMT, and PM were supported by the National Ecological Observatory Network, a program sponsored by the National Science Foundation and operated under cooperative agreement by Battelle.

Authors' contributions

RM drafted the original text with detailed input from the Omic BON co-leads (ND, PLB) and the Exploratory Committee (KJP, CM, RS), and in broad consultation with all the co-authors. All authors read and approved the final manuscript.

Acknowledgements

Any use of trade, firm, or product names is for descriptive purposes only and does not imply endorsement by the U.S. Government. The scientific results and conclusions, as well as any views or opinions expressed herein, are those of the author(s) and do not necessarily reflect those of OAR or the Department of Commerce.

Authors' information (optional)

Not applicable

References

1. Bourlat SJ, Borja A, Glibert J et al. Genomics in marine monitoring: New opportunities for assessing marine health status. *Mar Pollut Bull.* 2013;74:19–31. https://doi.org/10.1016/j.marpolbul.2013.05.042

2. Beale DJ, Jones OAH, Bose U et al. Omics-based ecosurveillance for the assessment of ecosystem function, health, and resilience. *Emerg Top Life Sci*. 2022;6:185–199. https://doi.org/10.1042/ETLS20210261

3. Díaz S, Settle J, Brondízio E. Summary for policymakers of the global assessment report on biodiversity and ecosystem services of the Intergovernmental Science-Policy Platform on Biodiversity and Ecosystem Services. 2019. IPBES secretariat. https://doi.org/10.5281/zenodo.3553579

4. Halpern BS, Longo C, Hardy D et al. An index to assess the health and benefits of the global ocean. *Nature*. 2012;488:615–620. https://doi.org/10.1038/nature11397

5. Wilkinson MD, Dumontier M, Aalbersberg IJJ et al. The FAIR Guiding Principles for scientific data management and stewardship. *Sci Data*. 2016;3:160018. https://doi.org/10.1038/sdata.2016.18

6. Carroll SR, Garba I, Figueroa-Rodríguez OL et al. The CARE Principles for Indigenous Data Governance. *Data Sci J.* 2020;19:43. https://doi.org/10.5334/dsj-2020-043

7. Aubertin C, Nivart A. Nature in Common: Beyond the Nagoya Protocol. *IRD Éditions*. 2021. ISBN : 978-2-8565-3958-3

8. Omic BON. [cited 9 Dec 2022]. Available: https://geobon.org/bons/thematic-bon/omic-

bon/

9. Davies N, Field D, Amaral-Zettler L et al. The founding charter of the Genomic Observatories Network. *GigaSci.* 2014;3:2047–217X–3–2. https://doi.org/10.1186/2047-217X-3-2

10. Mc Cartney AM, Head MA, Tsosie KS et al. Indigenous peoples and local communities as partners in the sequencing of global eukaryotic biodiversity. *npj biodivers*. 2023;2:8. https://doi.org/10.1038/s44185-023-00013-7

Figure 1

Click here to access/download;Figure;Figure 1 Omic BON founding charter.pdf

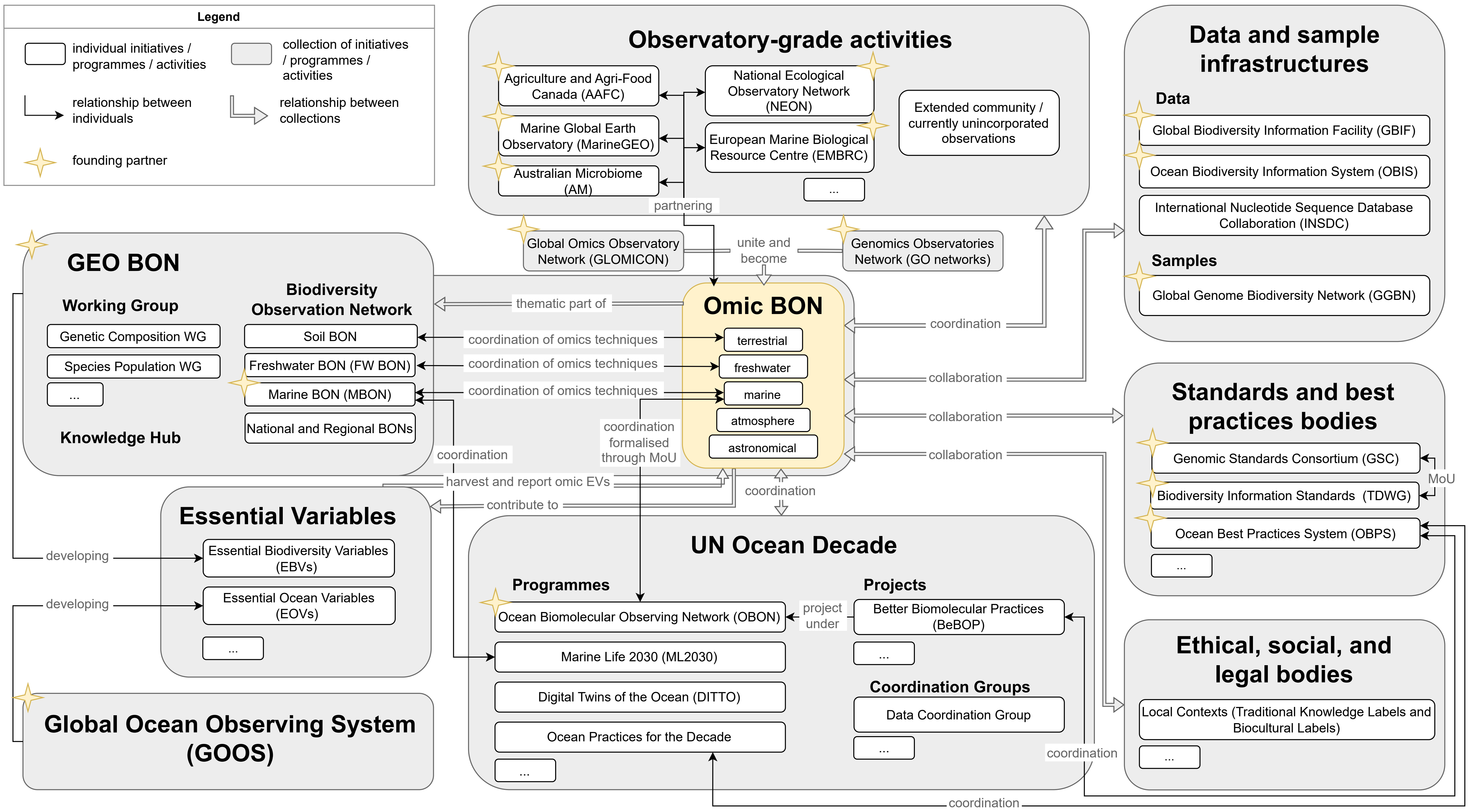

Figure 2 Click here to access/download;Figure;Figure 2 Omic BON founding charter.pdf

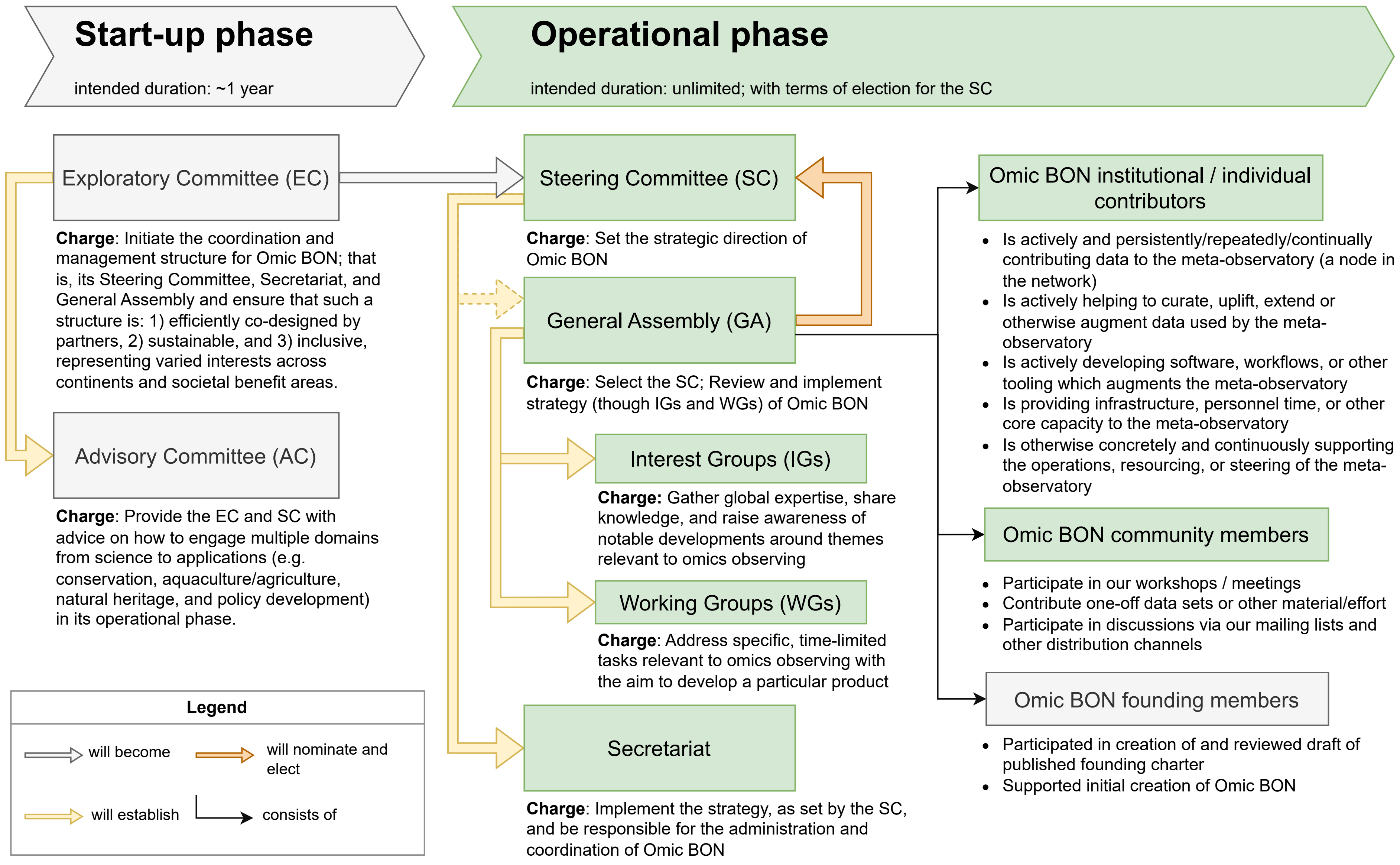

Cover Letter Click here to access/download;Personal Cover;Cover Letter.pdf

Raïssa Meyer
℅ Max Planck Insitute for Marine Microbiology
Celsiusstraße 1, DE-28359 Bremen
+49 421 2028-8670
raissa.meyer@awi.de

8th March 2023

Dear GigaScience editorial team,

The Editor of Gigascience Scott Edmunds has encouraged us to submit the commentary article titled "The Founding Charter of Omic Biodiversity Observation Network (Omic BON)" for publication in GigaScience.

The Omic Biodiversity Observation Network (Omic BON) is a thematic Biodiversity Observation Network (BON) under GEO BON[1], bringing together partners from national-, continental-, and global-scale observing systems, standards organisations, and data and sample infrastructures. Together we aim to establish a sustainable, responsive, and globally integrated omic meta-observatory that monitors biodiversity at the molecular level.

In the commentary, we present key elements of Omic BON's founding charter, introducing the first wave of activities to enhance molecular-scale biodiversity observations and transform them into actionable knowledge at a global scale.

We believe that the publication of this important commentary in GigaScience will provide a valuable platform for disseminating the ideas and goals of Omic BON to the scientific community. We appreciate the interdisciplinary nature of GigaScience and believe that the journal's broad reach and expert editorial board make it an ideal venue for this submission. Further, we value the continuity that comes with publishing Omic BON's founding charter in the same journal as that of one of its founding partners (Genomic Observatories network[2]).

The article represents original work, has not been published previously, and has not been submitted for publication elsewhere while under consideration. All authors have reviewed and approved the final version submitted for review, and there are no competing interests in relation to the work described.

Thank you for considering our submission. We look forward to the opportunity to contribute to the scientific discourse in GigaScience.

Yours sincerely,

Raïssa Meyer and Neil Davies - on behalf of all co-authors

---

[1] https://geobon.org/
[2] https://doi.org/10.1186/2047-217x-3-2
