# Supplementary material for: The founding charter of the Omic Biodiversity Observation Network (Omic BON)
Source: Gigascience. 2023 Aug 26;12:giad068. doi: 10.1093/gigascience/giad068 (PMC10460158; doi:10.1093/gigascience/giad068)
Supplement: giad068_Response_to_Reviewer_Comments_Original_Submission [file giad068_response_to_reviewer_comments_original_submission.pdf]

Author's Response To Reviewer Comments

Close

Dear Nicole Nogoy and Melody Clark,

Thank you for your positive response to our manuscript "The founding charter of the Omic Biodiversity Observation Network (Omic BON)".

I am thrilled to hear that you are considering it for publication in GigaScience and have made the requested formatting revisions to the references. Please let me know if any further changes are required.

I am delighted to hereby submit the revised version of the manuscript.

With thanks and regards,
Raïssa Meyer

Close
